# Development of Agrobacterium-Mediated Virus-Induced Gene Silencing and Performance Evaluation of Four Marker Genes in *Gossypium* *barbadense*

**DOI:** 10.1371/journal.pone.0073211

**Published:** 2013-09-02

**Authors:** Jinhuan Pang, Yue Zhu, Qing Li, Jinzhi Liu, Yingchuan Tian, Yule Liu, Jiahe Wu

**Affiliations:** 1 Institute of Microbiology, Chinese Academy of Sciences, Beijing, China; 2 State Key Laboratory of Cotton Biology of CRI, CAAS, Anyang, Henan, China; 3 MOE Key Laboratory of Bioinformatics, School of Life Sciences, Tsinghua University, Beijing, China; National University of Singapore, Singapore

## Abstract

*Gossypium* *barbadense* is a cultivated cotton species and possesses many desirable traits, including high fiber quality and resistance to pathogens, especially *Verticilliumdahliae* (a devastating pathogen of *Gossypium hirsutum*, the main cultivated species). These elite traits are difficult to be introduced into *G. hirsutum* through classical breeding methods. In addition, genetic transformation of *G* *. barbadense* has not been successfully performed. It is therefore important to develop methods for evaluating the function and molecular mechanism of genes in *G* *. barbadense* . In this study, we had successfully introduced a virus-induced gene silencing (VIGS) system into three cultivars of *G* *. barbadense* by inserting marker genes into the tobacco rattle virus (TRV) vector. After we optimized the VIGS conditions, including light intensity, photoperiod, seedling age and Agrobacterium strain, 100% of plants agroinfiltrated with the *GaPDS* silencing vector showed white colored leaves. Three other marker genes, *GaCLA1*, *GaANS* and *GaANR*, were employed to further test this VIGS system in *G* *. barbadense* . The transcript levels of the endogenous genes in the silenced plants were reduced by more than 99% compared to control plants; these plants presented phenotypic symptoms 2 weeks after inoculation. We introduced a fusing sequence fragment of *GaPDS* and *GaANR* gene silencing vectors into a single plant, which resulted in both photobleaching and brownish coloration. The extent of silencing in plants agroinfiltrated with fusing two-gene-silencing vector was consistent with plants harboring a single gene silencing vector. The development of this VIGS system should promote analysis of gene function in *G* *. barbadense* , and help to contribute desirable traits for breeding of *G* *. barbadense* and *G. hirsutum*.

## Introduction

Virus-induced gene silencing (VIGS) vector technology exploits the plant defense system against virus RNA. The dsRNA replication intermediate is processed into small interfering RNAs (siRNA) in the infected cell that correspond to parts of the viral vector genome, including any nonviral insert [1]. When a partial fragment of a candidate gene is inserted into a virus vector, plants inoculated with the recombinant virus generate virus-related siRNAs [2]. These siRNAs can mediate degradation of related endogenous gene transcripts, resulting in silencing of target gene expression [3,4]. Thus, VIGS can be used to investigate gene function as an alternative to plant transformation [4,5]. Nowadays, VIGS has become an important reverse genetics tool for gene function studies and functional genomics in various higher plants, such as tobacco [6,7], Arabidopsis [8], tomato [9], barley [10], soybean [11], *Medicago truncatula* [12], poppy [13] and the shrub *Jatropha* [14]. Recently, there have been reports showing that different VIGS systems are effective in *Gossypium hirsutum*. Tuttle et al. [15] reported the silencing of two visible marker genes, magnesium chelatase subunit I and phytoene desaturase (*PDS*), using the geminivirus *Cotton leaf crumple virus* (CLCrV) as a vector. Gao et al. [16,17] used the Tobacco rattle virus (TRV) as a vector to silence another marker gene, cloroplastos alterados 1 (*CLA1*). Although both VIGS systems were shown to work in *G. hirsutum*, it was not known whether these methods could be used to assess gene function in *G* *. barbadense* .

Cotton ( *Gossypium* spp.) is an important economic crop throughout the world because its fibers support one of the world’s largest industries, textiles. In addition, cotton is also an important source of foodstuffs, feed, oil, and biofuels [18]. Of about 50 species in the genus *Gossypium* , only four are cultivated in agriculture, including two allotetraploids, *G. hirsutum* and *G* *. barbadense* , and two diploids, *G* *. herbaceum* and *G* *. arboretum* [19,20]. At present, cotton cultivars mainly come from the two allotetraploids; few diploid cultivars are planted around the world. Although *G* *. barbadense* accounts for only 5% of cotton crops by area, it has many desirable traits that *G. hirsutum* lacks, such as excellent fiber quality (mainly length and strength of the fiber) and high tolerance/resistance to abiotic and biotic stresses, especially to Verticillium wilt disease, which causes devastating damage to *G. hirsutum*. Much effort has been expended to improve the fiber quality and tolerance/resistance of *G. hirsutum* by introducing desirable traits from *G* *. barbadense* using classic cross-fertilization, but it has not been successful because of the complex genetic backgrounds of the two species. On the other hand, although transgenic Bt and herbicide-resistant cotton cultivars have been successfully commercialized around the world for control of pests and weeds, large-scale gene isolation and functional analysis has lagged behind other agricultural plant species, such as rice, corn and oilseed. The main reasons for this are the large genome size, polyploidy, gene duplication, a long growth cycle, and recalcitrance to genetic transformation. To date, progress in genetic transformation of cotton has been made in *G. hirsutum*, but no reports of successful *G* *. barbadense* transformation have been presented. Thus, it is essential to develop molecular tools and resources for large-scale analysis of gene function in *G* *. barbadense* to further isolate its elite traits. Ultimately, understanding the molecular mechanisms of gene function and regulation will enable improvement of both *G* *. barbadense* and *G. hirsutum* through genetic engineering and molecular breeding.

In this study, we developed the first VIGS system in *G* *. barbadense* using TRV as a vector. Four marker genes, *CLA1*, *PDS*, *ANS*, and *ANR*, were silenced via this system with 100% efficiency under optimized conditions. In addition, a fusing two-gene-silencing vector containing *PDS and ANR* was agroinfiltrated into a single plant, both photobleaching and brownish coloration was apparent, in which the extent of silencing was as well as plants injected with a single gene silencing vector. However, when two marker gene vectors were mixed and simultaneously silenced in one plant, the symptoms were not well shown and the extent of silencing was reduced by interference. Our results will promote analysis of gene function in *G* *. barbadense* which help exploit desirable genes from this species.

## Materials and Methods

### Plant materials

Three *G* *. barbadense* cultivars, 3-79, Pima 90-53 and Hai 7124, were kindly supplied by Professor Luo Xiaoli from the Institute of Cotton Research, Shanxi Agricultural Academy of Science. Seeds were germinated and grown in a greenhouse. Seedlings from cotyledon expansion to 4^th^ true leaf emergence were used for VIGS assays. Infiltrated plants were grown in a growth chamber at 25°C with a 16/8 h light/dark photoperiod.

### Gene cloning and generation of recombinant VIGS vectors

Cotton gene sequences were obtained from the EST database of GenBank. Candidate gene fragments were cloned by PCR and inserted into the pYL156 vector [9] to generate pYL156 derivatives. The gene fragment sequences, fusing gene fragment sequences, and primers for cloning are listed in File S1 and Table S1, respectively.

Plasmids containing the binary TRV vectors, pTRV-RNA1, pYL156 and the pYL156 derivatives, were transformed into *Agrobacterium tumefaciens* strains GV3101, LBA4404 and EHA105 by electroporation.

### *Agrobacterium* infiltration

Agrobacterium cultures were grown overnight at 28°C in LB medium containing antibiotics (50 µg/ml kanamycin, 25 µg/ml gentamicin), 10 mM MES and 20 µM acetosyringone. The cells were pelleted by centrifugation at 1200 × *g* at room temperature for 8 min, and resuspended in MMA (10 mM MES, 10 mM MgCl_2_, 200 µM acetosyringone) solution to a final OD_600_ of 1.5. Cell suspensions were incubated at room temperature for at least 3 h without shaking. Agrobacterium cultures containing pTRV-RNA1 and pYL156 or its derivatives were mixed at a 1:1 ratio and infiltrated into two fully expanded cotyledons or true leaves using a needleless syringe. To facilitate the infiltration, small holes were punched with a needle on the underside of the cotyledons or leaves. We used separate pots for different TRV treatments to ensure that treated plants were not contaminated by a different TRV vector. After completion of the experiment, the plants and soil were autoclaved before disposal to prevent release of the virus into the environment. VIGS experiments were repeated three times, with eight plants for each construct per repeat.

### Optimization of conditions for TRV VIGS in *G. barbadense*

The agroinfiltrated plants were divided into three groups and grown under different light intensities, 100 µmol m^-2^ s^-1^, 300 µmol m^-2^ s^-1^ and 500 µmol m^-2^ s^-1^, to evaluate the effect of light intensity on photobleaching due to *PDS* silencing. Three different photoperiods, 16/8, 12/12 and 8/16 h (day/night), were evaluated using the same experimental procedure as above. The leaves of plants at different ages (cotyledon, 1^st^ leaf, 2^nd^ leaf, 3^rd^ leaf, 4^th^ leaf, and 5^th^ leaf stages) were agroinfiltrated to test the effect of *PDS* silencing. To evaluate the effect of Agrobacterium strain and culture concentration on the photobleaching of leaves by *PDS* silencing, three strains of Agrobacterium, GV3101, LBA4404 and EHA105, were infiltrated into plants at four concentrations (OD_600_ values 0.5, 1.0, 1.5 and 2.0). Sixteen plants formed a group in these experiments, which were repeated three times. Data were analyzed by one-way ANOVA in SAS [21]. Means were compared using Fisher’s least significant difference (LSD) test [21].

### RNA extraction and quantitative real-time PCR (qPCR) analysis

Total RNA was extracted from 100 mg fresh leaves using Trizol reagent according to the manufacturer’s instructions (Invitrogen, Carlsbad, CA, USA) and treated extensively with RNase-free DNase I (Promega, Madison, WI, USA). First-strand cDNA was synthesized from 2 µg total RNA with the SuperScript^TM^ First-Strand Synthesis system (Invitrogen). To further quantify target gene expression in the transgenic plants, qPCR was employed. Real-time PCR assays were performed using the SYBR Green Real-Time PCR Master Mix (Toyobo, Osaka, Japan) and the DNA Engine Opticon 2 Real-Time PCR Detection System (MJ Research, Hercules, CA, USA). The cotton *GhUBI* gene was used as an internal control to normalize expression. For each construct, three plants with obvious phenotype were sampled. All reactions were performed in triplicate; negative controls were run without reverse transcriptase. Primer sequences are listed in Table S1. Data were processed to determine relative transcript abundances using the Opticon Monitor software (Bio-Rad, Hercules, CA, USA).

To measure changes in TRV virus biomass, relative quantification of the TRV RNA2 included in the pYL156 vector was performed. Total RNA of systemic leaves from plants inoculated with pYL156 and its derivatives was isolated, cDNA was synthesized and qPCR was carried out as described above. The primers for TRV RNA2 are listed in Table S1. The cotton *GhUBI* gene was used as an internal control to normalize expression.

### Chlorophyll and histochemical assay

Total leaf chlorophyll content was measured 14 d after plants were inoculated with silencing vectors according to the method described by Tang et al [22].

Proanthocyanidin (PA) staining was carried out using the DMACA (p-dimethylaminocinnamaldehyde)-HCl protocol described by Li et al [23]. Fresh leaves were immediately soaked in ethanol-glacial acetic acid solution (3:1, v/v) for decoloration. The decolorized leaves were stained for about 20 min with 0.3% (w/v) DMACA in a cold mixture of methanol and 6 M HCl (1:1, v/v), and then rinsed with several changes of 70% (v/v) ethanol. PA containing cells were stained blue.

## Results

### Optimal conditions for TRV VIGS in G. barbadense

**Figure 1:**
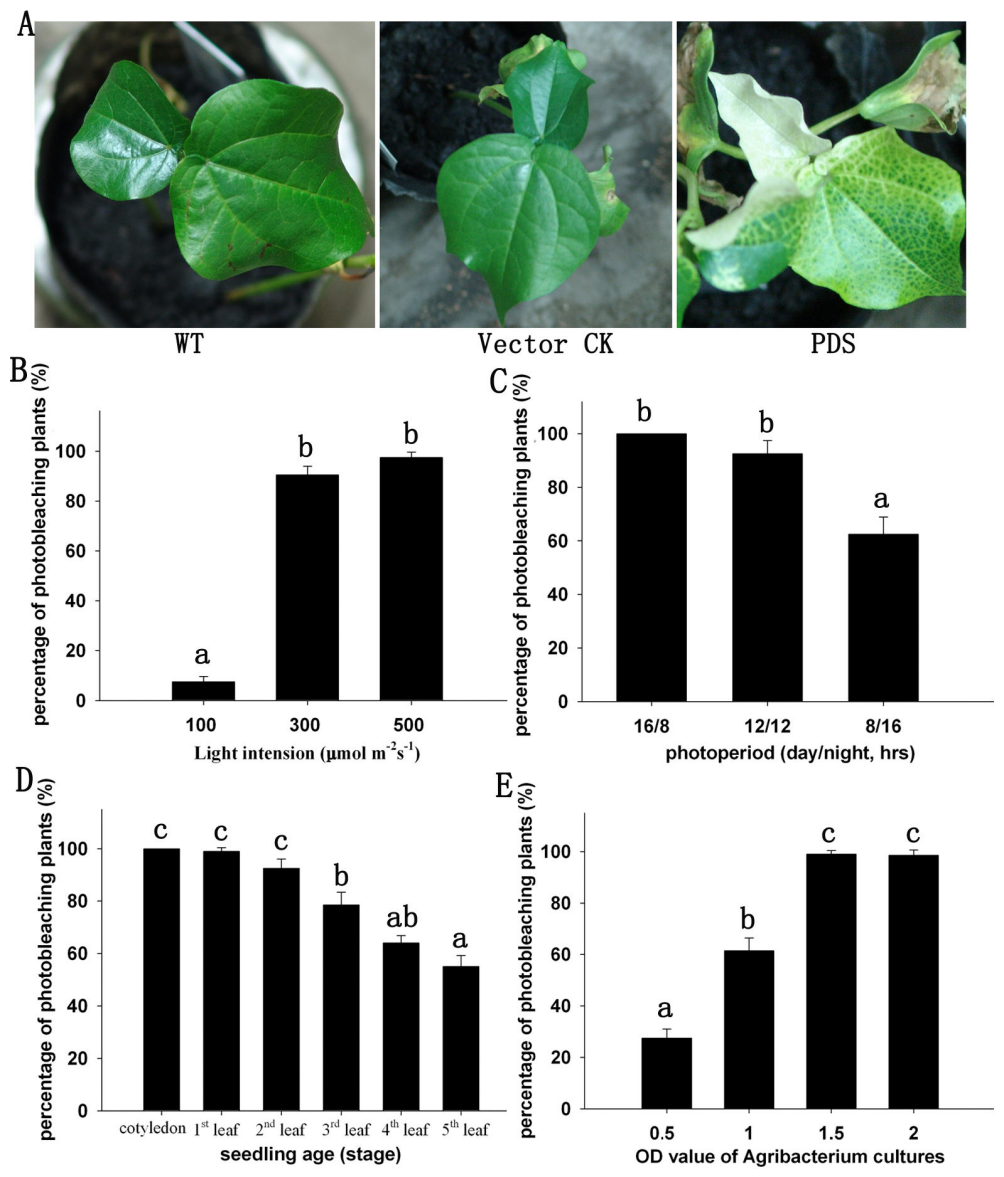
Optimal factors for Agrobacterium-mediated *GaPDS* VIGS in *G* *. barbadense* . A, The photobleaching phenotype of cotton leaves triggered by *GaPDS* VIGS. The pYL156 vector was used as a vector control; WT, wild type. B–E, The percentage of plants showing photobleaching was affected by light intensity, photoperiod, seedling age, and OD value of Agrobacterium cultures. Means ± standard deviation labeled with different letters are significantly different at the 0.05 level.

In our initial experiment to develop an Agrobacterium-mediated VIGS system in *G* *. barbadense* , we investigated the optimal conditions under which TRV-based VIGS in *G* *. barbadense* cv 3-79 was effective by attempting to silence the *G* *. barbadense* Phytoene desaturase (GaPDS) gene as a marker. *PDS* has been used as a marker for the effectiveness of VIGS in several instances [8,9,24]. The silencing of *PDS* typically results in white leaves caused by photobleaching, which occurs in the absence of the gene product. In addition, the VIGS vector tobacco rattle virus (TRV) is able to spread vigorously throughout the entire plant but produces only mild symptoms [4]. *G* *. barbadense* gene sequences with homology to Arabidopsis genes encoding PDS were used for functional analysis. Partial fragments of putative cotton *PDS* were cloned into the pYL156 vector to give pYL156:*PDS*. Plants infiltrated with the empty pTRV-RNA vector (pTRV-RNA1 + pYL156) were used as controls in this study. All plants infiltrated with empty vector (henceforth referred to as vector control plants) grew normally and showed normal phenotypes (Figure 1A). No phenotypic differences were observed between uninfected plants and vector control plants (Figure 1A); this is one of the important advantages of TRV-mediated gene silencing.

Next, we began to optimize factors for TRV VIGS in *G* *. barbadense* . We compared the number of plants showing the *PDS*-silenced phenotype after growth under different light intensities and photoperiods. Among the seedlings grown under high light conditions (300 and 500 µmol m^-2^ s^-1^), 90% and 97.5% of plants, respectively, displayed photobleaching at 2 weeks post-inoculation, compared with only 8% of those grown under low light (100 µmol m^-2^ s^-1^) (Figure 1B). For different photoperiods, the number of plants showing symptoms of leaf photobleaching under long-day conditions was significantly higher than those grown under short-day conditions. Under a 16/8 h photoperiod, 100% of plants displayed white leaves, compared to 92.5% and 62.5%, respectively, of those grown under 12/12 and 8/16-h photoperiods (Figure 1C).

After testing seedlings of different ages, we found that silencing of *PDS* was effective in seedlings inoculated from the expanding cotyledon stage to the one- to two-leaf stage, 90–100% of which exhibited photobleaching symptoms. The number of plants displaying the *PDS*-silenced phenotype decreased by 55% when seedlings at the five-leaf stage were injected (Figure 1D). Therefore, younger plants were better for TRV-based silencing in *G* *. barbadense* . In addition, the extent of leaf photobleaching was greater by inoculating cotyledons rather than true leaves.

We also investigated whether the strain and culture concentration of *A. tumefaciens* used to introduce the VIGS vectors affected the outcome of silencing in *G* *. barbadense* . The three strains of *A. tumefaciens* tested, GV3101, LBA4404 and EHA105, showed no significant difference in the number of the plants with the VIGS phenotype (data not shown). However, the concentration of the cultures used for agroinfiltration had a significant effect on silencing of *PDS*. Cultures resuspended at OD_600_ = 1.5 or above exhibited the greatest effect on VIGS in *G* *. barbadense* (Figure 1E).

Thus, *G* *. barbadense* seedlings inoculated at the cotyledon expansion to two leaf stages and grown under a 300 µmol m^-2^ s^-1^ or greater light intensity with a photoperiod of at least 12-h light displayed the photobleaching phenotype indicative of *PDS* silencing in almost 100% of cases. We employed three endogenous cotton genes, *GaPDS*, *GaCLA* and *GaANR* to further evaluate the VIGS system in *G* *. barbadense* using these optimized conditions for agroinfiltration.

### GaPDS and GaCLA1 genes provide visible markers for endogenous gene silencing

VIGS has proven to be a powerful tool for gene function studies and functional genomics in various higher plants including *G. hirsutum*. Having optimized the conditions for Agrobacterium-mediated VIGS in *G* *. barbadense* , we next evaluated the VIGS system for analysis of gene function by employing different endogenous marker genes.

**Figure 2:**
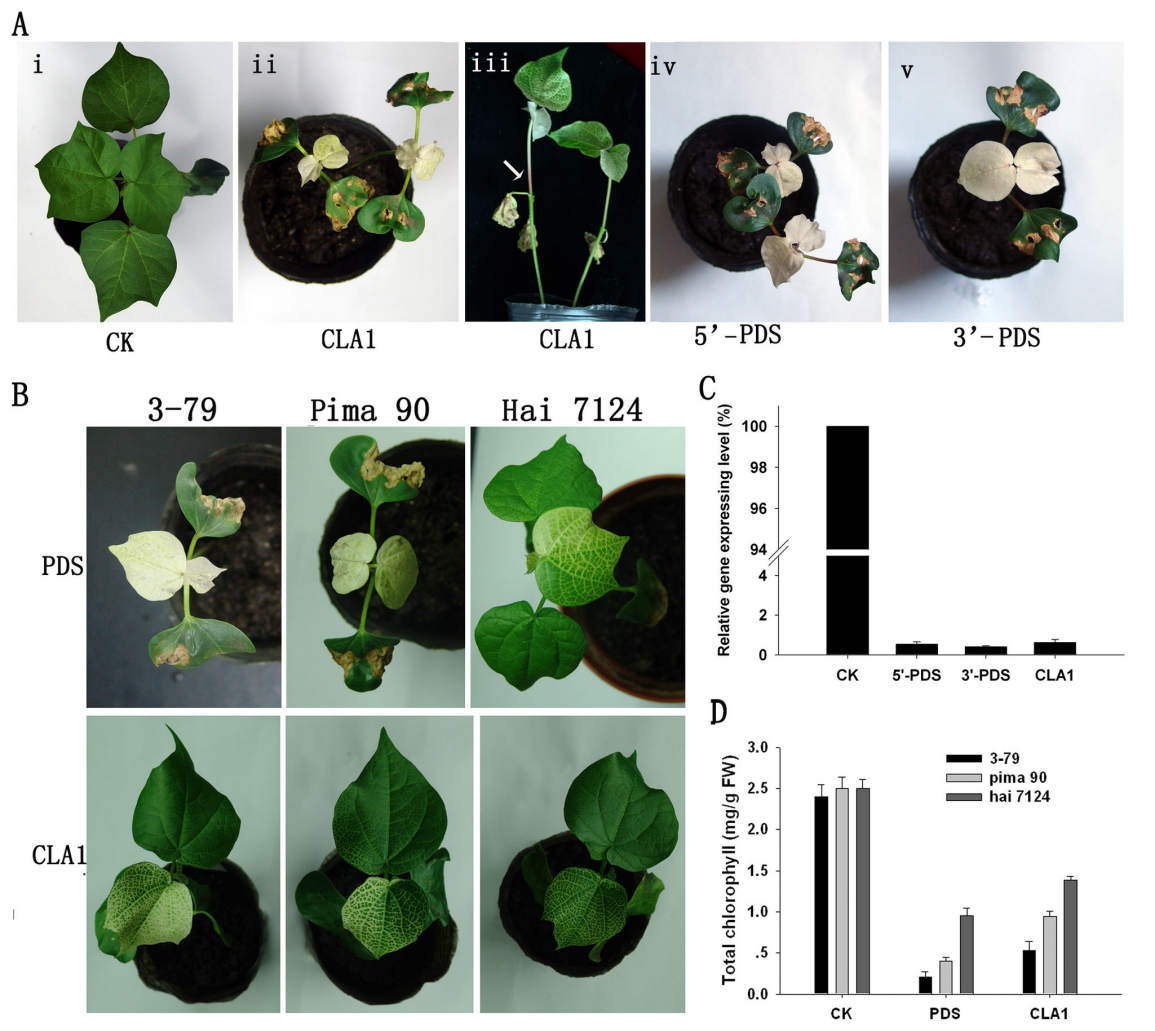
Agrobacterium-mediated TRV VIGS of two marker genes, *GaPDS* and *GaCLA1*, in *G* *. barbadense* . A, Phenotypes of plants inoculated with pYL156:*CLA1* or pYL156:*PDS* vectors. The pYL156 vector was used as a vector control. B, Three cotton cultivars exhibited the photobleaching phenotype triggered by *GaPDS* or *GaCLA1* gene silencing to differing extents. C, Relative transcript levels of *PDS* and *CLA1* in systemic leaves of plants infiltrated with pYL156:*PDS* or pYL156:*CLA1*. The CK value was set at 100%. D, Total chlorophyll content in photobleached leaves. Error bars represent standard deviations (n = 3 biological replicates) in (C) and (D).

The Cloroplastos alterados 1 gene (*CLA1*) has been used as a marker gene to investigate the VIGS system in many plants. Estevez et al. [25] reported that Arabidopsis* CLA1* encodes 1-deoxyxylulose 5-phosphate synthase, the first enzyme of the 2-C-methyl-d-erythritol-4-phosphate pathway involved in chloroplast development. We isolated a homolog of *CLA1* from *G* *. barbadense* , named *GbCLA1*. Initiation of a photobleaching phenotype in plants infiltrated with Agrobacteria expressing a partial sequence of *GbCLA1* was observed on newly emerging leaves at approximately 8–10 days post-infiltration. Twenty days later, 100% of these plants showed a photobleaching phenotype uniformly distributed across all true leaves (Figure 2A ii). The stems of these plants turned pink or purple, and the plants became severely stunted by the late growth stage (Figure 2A ii, iii), probably due to the strong inhibition of chlorophyll biosynthesis. The vector control plants did not display a photobleaching phenotype and grew normally (Figure 2A i).

The *PDS* gene described above is another commonly used marker gene for VIGS and also causes loss of chlorophyll and carotenoids [26,27]. The general pattern of cells and tissues with *GaPDS* silencing was similar to that of *GaCLA1*, but the extent of silencing was even greater. The leaves of *GaPDS*-silenced plants were all white, whereas pale yellow leaves were observed in *GaCLA1*-silenced plants (Figure 2A, B). To determine whether fragments from different regions of the gene affected the extent of silencing, two *GaPDS* inserts were amplified near the 5ʹ- and 3ʹ- termini (File S1). The results showed that the extent of silencing achieved was similar for both fragments (Figure 2A iv, v).

To verify the reduction in *GaCLA1* or *GaPDS* mRNA in silenced leaves, a different region of each target gene to that used in the vector was amplified from silenced tissues by qPCR. We collected leaf samples from three independent cotton plants with prominent phenotypes (based on the visible phenotype) for each gene and further analyzed the transcript levels in systemic leaves. We found that *CLA1* and *PDS* transcript levels were reduced by more than 99% in silenced plants compared with vector control plants (Figure 2C). These results from the pYL156:*CLA1* and pYL156:*PDS* plants confirmed the putative functions of these two cotton genes.

To date, a transformation system for *G* *. barbadense* has not been developed, so the ability to silence genes readily in *G* *. barbadense* using VIGS as a tool for gene function studies and functional genomics in cotton is desirable. Thus, we further tested the VIGS efficiency on three commercial cultivars that were grown in China, including 3-79, Pima 90-53 and Hai 7124. The photobleaching phenotype was observed in all three cultivars infiltrated with *GhPDS*-expressing agrobacteria within two weeks after inoculation (Figure 2B). Twenty days later, the photobleaching phenotype had uniformly spread across the entire leaves. The silencing efficiency in all cultivars tested was equally high and consistently reached 100% in multiple independent biological trials. However, for cultivar Hai 7124, the photobleaching phenotype was incomplete, with some green color remaining, although the silencing efficiency was 100% at 20 d after infiltration. Similarly, infiltration of *GhCLA1*-expressing agrobacteria triggered silencing of *GhCLA1* and the extent of photobleaching was greater in 3-79 and Pima 90-53 than in Hai 7124 (Figure 2B). The chlorophyll contents were assayed to determine the extent of photobleaching in leaves. Leaves of cultivar 3-79 were more heavily decolorized than those of the other two cultivars 14 d after inoculation with pYL156:*PDS* or pYL156:*CLA1* (Figure 2D). Taken together, these data suggest that a TRV-based VIGS system could trigger potent silencing of endogenous genes in *G* *. barbadense* plants, which are amenable to VIGS experiments for gene loss-of-function studies.

### The PA metabolic genes ANS and ANR provide mild marker genes for endogenous gene silencing by VIGS

*CLA1* and *PDS* provided obvious markers for analysis of gene and genome functions by the VIGS system, but plants in which these two genes were silenced died 1–2 months post inoculation because of chloroplast damage and cessation of photosynthesis. Thus, it is necessary to select marker genes that allow plants to grow to maturity for analyzing gene function with the VIGS system, especially for reproduction related genes. PAs are a major class of flavonoids, one of the largest groups of plant secondary metabolites. Most genetic studies on the PA biosynthesis pathway have been performed in Arabidopsis and *Medicago truncatula* [28,29]. The enzymes ANS and ANR function at branches between anthocyanin and PA biosynthesis. ANS converts the substrate flavan-3,4-diol (leucoanthocyanidin) to anthocyanidin, which serves as a substrate for ANR to produce a major PA, 2,3-cis-flavan-3-ol (epicatechin), in Arabidopsis and *Medicago* [28,29]. When the expression of these two proteins was repressed in mutants or interruptible plants, plants with lower PA content were able to grow and mature, and could be observed via phenotype and staining with the aromatic aldehyde agent DMACA.

**Figure 3:**
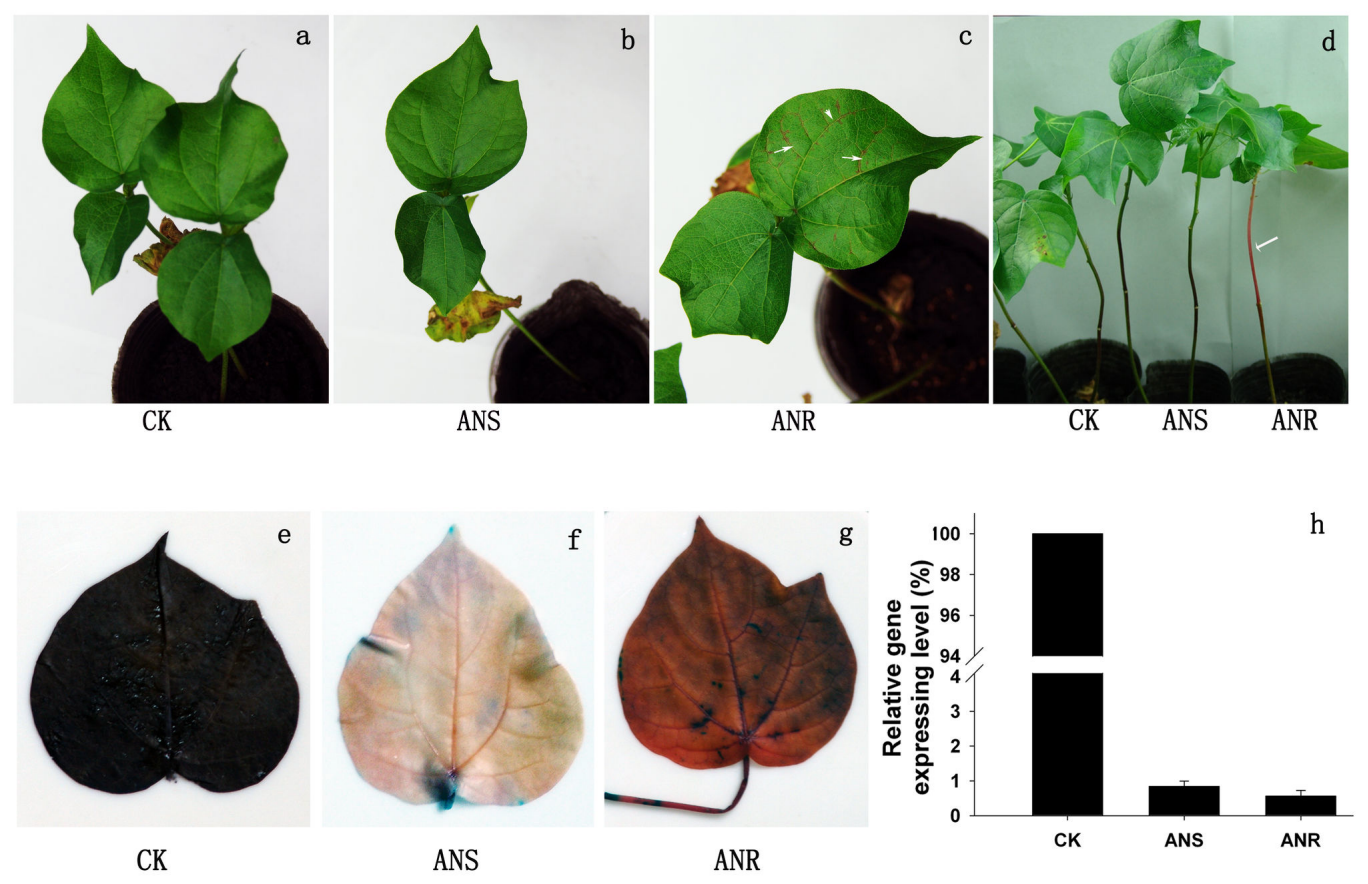
TRV-induced silencing of the anthocyanidin and proanthocyanidin biosynthetic genes *ANS* and *ANR*. a–d, Plants infiltrated with the vector control (CK), pYL156:*ANS* and pYL156:*ANR* showed different phenotypes in systemic leaves (a–c) and stems (d). e–g, DMACA stained leaves. h, Relative transcript levels of *ANS* and *ANR* in systemic leaves of plants infiltrated with pYL156:*ANS* and pYL156:*ANR*. The CK value was set at 100%. Error bars represent standard deviations (n = 3 biological replicates). White arrows indicate pink leaf veins (c) and stem (d).

Partial fragments of putative cotton *ANS* and *ANR* genes with homology to Arabidopsis genes were cloned into the pYL156 vector to generate pYL156:*ANS* and pYL156:*ANR*. No phenotypic differences were observed between uninfected plants and vector control plants. At 8–10 days post inoculation, leaf veins and leaf margins around new systemic leaves began to appear brownish in all of the pYL156:*ANR-*treated plants. One month later, leaves and leaf petioles (Figure 3C) and stems (Figure 3D) became brownish. This brownish phenotype could be due to anthocyanidin accumulation resulting from the loss of ANR function. In contrast, pYL156:*ANS* plants showed no visible phenotypic differences to vector control plants (Figure 3A, B, D). However, pYL156:*ANS* and control plants did show differences after PA staining with DMACA (Figure 3E, F). After staining, the leaves of vector control plants turned blue, indicating a high level of PA accumulation. Leaves from pYL156:*ANS* plants appeared pale yellow, indicating low anthocyanidin and PA levels, while leaves from pYL156:*ANR* plants appeared red, indicating anthocyanidin accumulation. Two months later, the brownish phenotype and staining markers could still be detected in organs, indicating that *GaANS* and *GaANR* could be used as mild marker genes for gene function analysis by VIGS. In addition, the cotton PAs were mainly insoluble in the cell; soluble PAs comprised about 5% of total PA content in OD_550_ assays. Both soluble and insoluble PAs decreased in systemic leaves of plants agroinfiltrated with pYL156:*ANS* or pYL156:*ANR* (Figure S1, File S2). We also analyzed the transcript levels of these genes in systemic leaves and found that *ANS* and *ANR* transcripts were reduced by more than 99% in silenced plants compared with vector control plants (Figure 3H).

### GaPDS and GaANR genes were simultaneously silenced in one plant by the VIGS system

**Figure 4:**
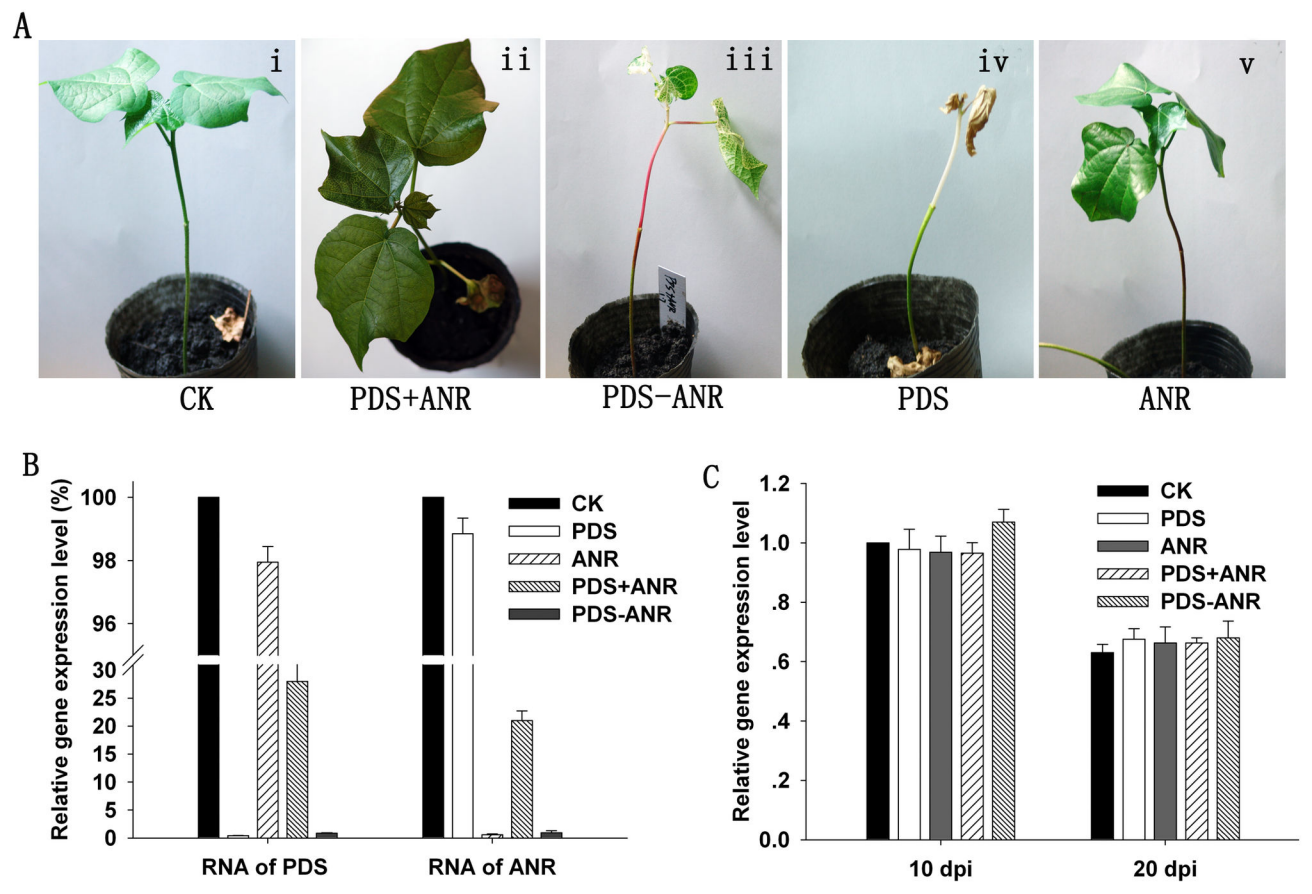
Simultaneous silencing of *GaPDS* and *GaANR* in a single plant with the VIGS system. A, The phenotypes of plants inoculated with pYL156 (i), pYL156:*PDS* + pYL156:*ANR* (ii), pYL156:*PDS-ANR* (iii), and pYL156:*PDS* (iv), and pYL156:*ANR* (v). B, Relative transcript levels of *PDS* and *ANR* in systemic leaves of plants infiltrated with pYL156:*PDS*, pYL156:*ANR*, and pYL156:*PDS* + pYL156:*ANR*, and pYL156:*PDS-ANR*. The CK value was set as 100%. C, Relative levels of TRV RNA2 in systemic leaves of plants infiltrated with pYL156:*PDS*, pYL156:*ANR*, and pYL156:*PDS* + pYL156:*ANR*, and pYL156:*PDS-ANR*. The CK value at 10 d post-inoculation (dpi) was set at 1. Error bars represent standard deviations (n = 3 biological replicates) in (B) and (C).

In plants, some proteins have the same or similar functions (for example isoenzymes) and some traits, such as resistance to biotic and abiotic stresses, can be determined by several genes. In such cases, the phenotype cannot be uncovered when only a single gene is silenced by VIGS. In addition, large-scale VIGS experiments have been adopted as a fast-forward genetics approach to screen for phenotypes of interest [4,30–32]. While this approach could be used for high-throughput screening of important genes in cotton, it takes considerable space and effort to grow a large number of cotton seedlings for VIGS. Considering these factors, we sought to determine whether one plant could be inoculated with fusing several-gene-silencing vector or mixing multiple gene-silencing vectors. We fused the sequence fragments of *GaPDS* and *GaANR* by chemical synthesis and inserted into pYL156 and generated pYL156:*PDS-ANR* vector. At 10 d after inoculation with pYL156:*PDS-ANR* vector, the two distinct symptoms, photobleaching and brownish coloration, were both obvious in the one plant. However, when we mixed three different Agrobacterium cultures containing pTRV-RNA1, pYL156:*PDS* and pYL156:*ANR* at a 2:1:1 ratio to inject a single plant, the symptoms were not well observed (Figure 4A).

Leaf samples from three independent cotton plants with prominent phenotypes were collected, and the transcript levels of *GaPDS* and *GaANR* were analyzed. We found both a 99% reduction in transcript level for these genes in the silenced plants with fusing two-gene-silencing vector compared with vector control plants (Figure 4B). However, the transcript levels of the two genes in one plant simultaneously inoculated with mixed pYL156: *PDS* and pYL156:*ANR* were obviously higher than in plants inoculated with either pYL156:*PDS* or pYL156:*ANR*. The reductions of transcript level were respectively 72% and 79% for *PDS* and *ANR* gene compared to vector control plants (Figure 4B). This result suggested that it is useful and feasible to simultaneously silence two genes in a single plant through inoculation with fusing two-gene-silencing vector, but not with mixing two gene-silencing vectors.

To test whether the differences in *GaPDS* and *GaANR* expression among plants agroinfiltrated with fusing two-gene-silencing vector, mixing two gene-silencing vectors, and a single gene-silencing vector were correlated with virus growth in the host tissue, measurements of virus RNA2 in the infected leaves were made. Total RNA was extracted from systemic leaves of plants inoculated with different vectors. Virus RNA2 and the cotton internal control gene *GaUBI* were then quantified by real-time PCR. At 10 and 20 days after inoculation, the amount of virus RNA2 was similar among all inoculated plants at each time point. However, the levels at 10 d post-inoculation (dpi) were higher than those at 20 dpi. This result indicated that the amount of virus in a cell was stable regardless of whether one or multiple gene silencing vectors were introduced by the TRV-based VIGS system. Taken together, these results indicated that under fusing these genes condition, two or three genes could be simultaneously silenced in one plant for analysis of gene and genome functions by TRV-based VIGS although the total virus amount in a cell was stable. Thus, we suggest that it would be useful and feasible to silence multiple genes in one plant via fusing these genes silencing vector. The genes identified in this VIGS system will provide valuable resources for targeted breeding programs and genetic engineering of cotton cultivars with desirable traits in *G* *. barbadense* .

## Discussion

Cotton is an important crop as a source of fibers and oil. Improving cotton yield and fiber quality is the main goal of cotton breeding. Biotechnology and molecular technology have been introduced into cotton cultivar improvement, and transformation and molecular marker techniques have been developed to assist cotton breeding. However, genetic transformation of cotton is laborious and technically challenging, so has limited utility for analysis of gene function and germ plasm innovation. In addition, genetic transformation methods have still not been successfully developed for *G* *. barbadense* , which has many desirable traits such as long fibers, high fiber strength and resistance to Verticillium wilt disease, and thus gene functions and molecular mechanisms in this species have not been dissected. New techniques need to be developed to promote genetic studies of cotton, especially *G* *. barbadense* . In the present study, we developed the first TRV VIGS system in *G* *. barbadense* . Four marker genes, *GaPDS*, *GaCLA1*, *GaANS*, and *GaANR*, were silenced using this system, producing measurable phenotypes. To date, there have been four papers reporting the use of gene silencing in *G. hirsutum* with different virus vectors. Gao et al. [16,17] showed that TRV-VIGS functions in cotton leaves with 100% silencing efficiency by targeting the *GhPDS* gene, Tuttle et al. [15] explored the geminivirus *Cotton leaf crumple virus* (CLCrV) as a vector to dissect the functions of genes by gene silencing, and Qu et al. [33] chemically synthesized TRV vectors to extend their utility to reproductive organs and tissues.

We optimized conditions for the TRV VIGS system in *G* *. barbadense* , including light intensity, photoperiod, seedling age, and Agrobacterium strain and culture concentration, by silencing the *GaPDS* gene. Our results suggested that high light intensity, a long-day photoperiod, inoculation of young cotyledons and an OD value of 1.5 for the Agrobacterium culture could promote the photobleaching phenotype triggered by *GaPDS* gene silencing. Gao et al. [16,17] selected plants with two fully expanded cotyledons to infiltrate with Agrobacterium culture using a syringe, and observed strong gene silencing by VIGS. However, Qu et al. [33] employed cotton plants with 2–4 true leaves for Agrobacterium-mediated VIGS via vacuum infiltration, and also observed that the silencing efficiency of marker genes reached 100%. Our results suggested that the efficiency of *PDS* gene silencing by syringe infiltration was higher using cotyledons rather than true leaves. When using chloroplast/chlorophyll synthesis-related genes as markers, e.g. *PDS*, *CLA1*, or *ChlI* (magnesium chelatase subunit I), high light intensity and a long-day photoperiod facilitate the appearance of the photobleaching phenotype. Most papers involving VIGS technology reported using light conditions that included a photoperiod of 12 h or more and a light intensity of over 300 µmol m^-2^ s^-1^ [6,9,10,15–17,25,33].

Most marker genes used in the VIGS system are related to chlorophyll synthesis because of their visible phenotype in plant leaves. However, there are limitations in using these marker genes to evaluate plant gene functions because of their short period of usefulness. When these marker genes, e.g. *PDS*, *CLA1* and *ChlI*, are silenced, photobleaching or chlorosis rapidly occurs in leaves. If functional leaves become photobleached due to failure of chlorophyll synthesis, the plants will die from a shortage of nutrients [6,9,16,17,25]. Therefore, mild marker genes should be explored for analysis of gene functions, especially for reproductive genes. Qu et al. [33] reported using *GhANR* as a marker to analyze the functions of genes involved in fiber development in *G. hirsutum*. In our study, *ANS* and *ANR* were also confirmed to be mild marker genes in *G* *. barbadense* , exhibiting phenotypes related to PA content, making them suitable markers for gene function research as the silenced plants can grow to maturity and set seeds. In addition, we found that the cotton PAs were mainly insoluble in the cell, with soluble PAs comprising about 5% of total PA content. Both soluble and insoluble PAs decreased in the systemic leaves of plants agroinfiltrated with pYL156:*ANS* or pYL156:*ANR* (Figure S1, File S2).

In our study, when *GaPDS* and *GaANR* were simultaneously agroinfiltrated in one plant with mixing two gene-silencing vectors, the two genes interfered with each other. The photobleaching and red leaf vein or stem phenotypes were not well apparent in plants (Figure 4A). The reduction in transcript level of the two target genes in the same plant was also lower than in plants with a single gene silenced (Figure 4B). however, under fusing the sequence fragments of *GaPDS* and *GaANR* gene condition, the extent of phenotypic change and transcript level reduction for these genes in plants simultaneously silenced two genes was the same as in plants silenced a single gene. Gao et al. [17] reported that VIGS still occurred effectively even when the Agrobacterium culture containing pTRV2-CLA1 was mixed with an Agrobacterium culture containing the pTRV2 control vector at a 1:49 ratio (50-fold dilution) for infiltration. However, our data did not support this notion when simultaneously silencing *GaPDS* and *GaANR* in one plant through mixing vector mode. We consider the data of Gao et al. [17] to be reliable; their results may be explained by the high sensitivity of the *CLA1* gene in the VIGS system. In another paper, Fu et al. [34] found that the TRV-vector was not transferred from one fruit to another or to other parts of the plant, which may therefore allow silencing of different genes in different fruits on the same plant. However, most studies on VIGS consider the silencing phenotype to be mediated by an intercellular signal that spreads from the infected cell [35–37]. This signal may be a nucleic acid, either double-stranded RNA or one of the classes of short RNA associated with silencing.

## Supporting Information

Figure S1 **Assay of soluble and insoluble PA(s).** A and C, The photographs of insoluble and soluble PAs from the systemic leaves of control vector, ANR silencing, and ANS silencing plants. B and D, the OD550 value of insoluble and soluble PAs. Error bars represent standard deviations (n= 3 biological replicates). 100mg leaves are employed, and the extraction was dissolved in 1 ml buffer to test OD550 value.
(TIF)

File S1 **Sequence information of gene fragment used for VIGS.**
(DOC)

File S2 **Extraction and assay of soluble and insoluble PA(s).**
(DOC)

Table S1 **Primer sets used to isolate target gene fragment sequences for PCR and assay for qRT-PCR.**
(DOC)
