# Supplementary figures and images for: Development of Agrobacterium-Mediated Virus-Induced Gene Silencing and Performance Evaluation of Four Marker Genes in Gossypium barbadense
Source: PLoS One. 2013 Sep 2;8(9):e73211. doi: 10.1371/journal.pone.0073211 (PMC3759462; doi:10.1371/journal.pone.0073211)

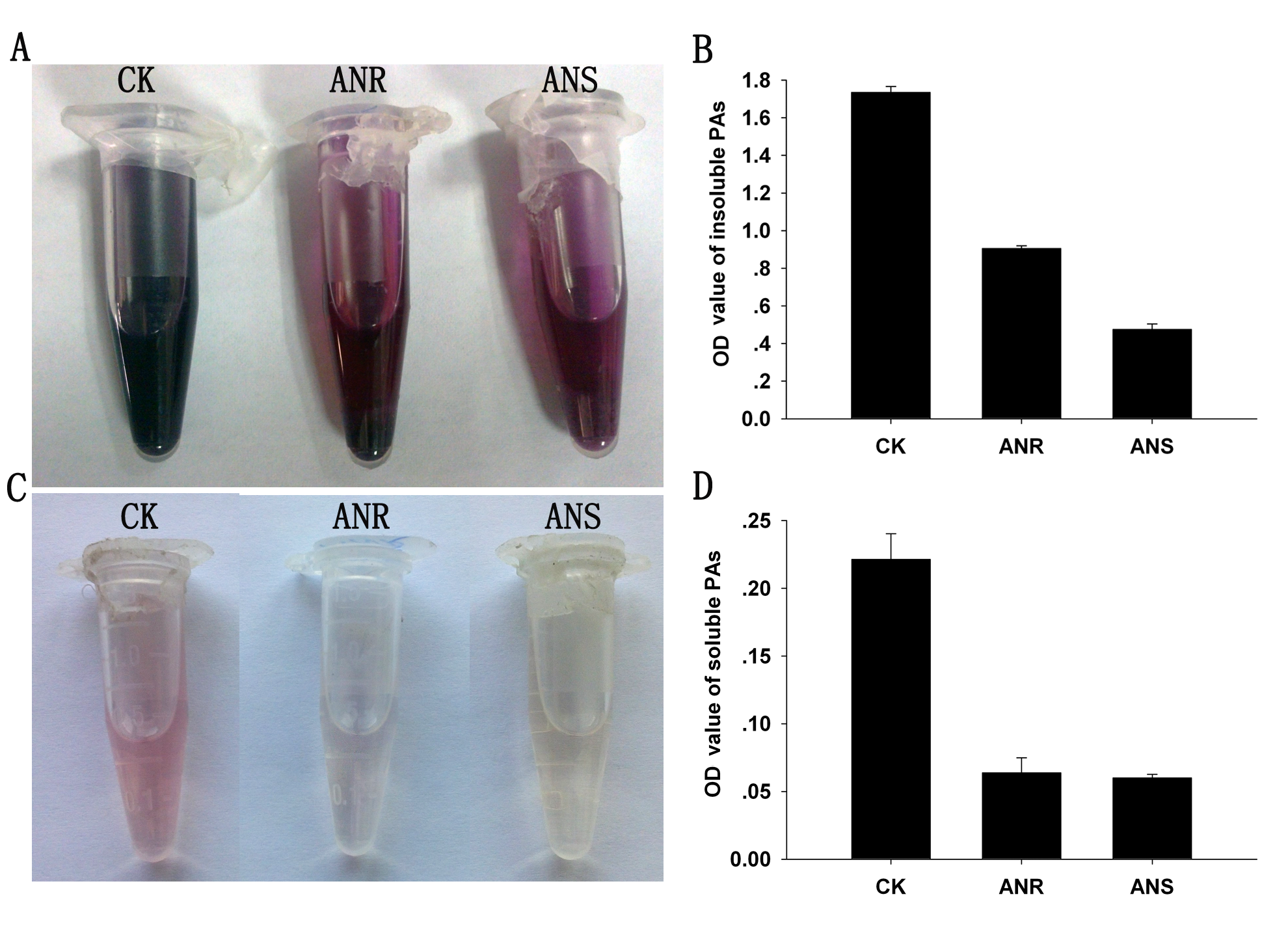

Supplement: Figure S1 — Assay of soluble and insoluble PA(s). A and C, The photographs of insoluble and soluble PAs from the systemic leaves of control vector, ANR silencing, and ANS silencing plants. B and D, the OD550 value of insoluble and soluble PAs. Error bars represent standard deviations (n= 3 biological replicates). 100mg leaves are employed, and the extraction was dissolved in 1 ml buffer to test OD550 value. (TIF) [file pone.0073211.s001.tif]
