# Supplementary material for: Development of Agrobacterium-Mediated Virus-Induced Gene Silencing and Performance Evaluation of Four Marker Genes in Gossypium barbadense
Source: PLoS One. 2013 Sep 2;8(9):e73211. doi: 10.1371/journal.pone.0073211 (PMC3759462; doi:10.1371/journal.pone.0073211)
Supplement: File S1 — Sequence information of gene fragment used for VIGS. (DOC) [file pone.0073211.s002.doc]

**File S1. Sequence information of gene fragment used for VIGS**

5’-GaPDS

tgcgatgccaaataaacctggagagttcagtcgatttgattttccagaagttctacctgcaccattaaatggaatatgggccattttgaagaataatgaaatgctgacttggccagagaaagtgaaatttgcaataggactcctaccagcaatgctgggtggacaaccttatgttgaggcccaagatggtttatctgttaaagactggatgagaaagcagggcgtacctgatcgtgtgactgaggaggtgt

3’-GaPDS

gcctgaagactggagagagatttcatacttcaagaaattagagaaattagttggagttccagttatcaacgttcacatctggtttgataggaaattgaagaacacctatgatcatctactctttagcagaagcccgcttttaagtgtttatgctgacatgtctgtaacatgtaaggaatattacaatccaaaccaatccatgttggagttagtttttgccccagcagaagaatggattgcatgtagtgactcagaaattattgatgctacaatgaaggaacttgcaaagctctttcctgatgaaatatctgcagatcagagtaaagc

GaCLA1

gccctttgtgcatcttcatttcctgccattatcaactggggtgcagcttcagatcctcaaaaatccactccttttgcttcccattttcttggtgg atcagatctggttttgcagccattgaagaagctcaatcaggtcaagaaaaggccaggtggggcttatgcatcactatcagaaggagctgaatatcactcccaaagaccagcaacgcctctcttggacactataaactatctaattcatatgaaaaatctctctgtcaaggaactgaaacaactatctgaagaactgcggtctgatgtggttttcaatgtttcaaaaactgggggtcacttgggttcaagccttggtgtggttgaactcactgtggctcttcattatgtcttcaatgcccctagag

GaANR

aacactacagtcagggaccctgacaaccagaagaagatctctcaccttgtaacactacaagagttgggagacttgaagatctttcaggcggatttaactgatgaagggagctttgatgcccctattgctggttgtgaccttgtcttccatgttgcgacacccgttaactttgcttctgaagatccagaggatgacatgatcaaaccagcgactcaaggagtggtgaacgttttgaaagcttgtgccaaagcaaaaacagttaaacgagtcgtcttgacatcatctgccgca

GaANS

gcttgagtgggaggactacttcttccatcttatttttcctgaggataagagagacttgtcaatttggcctaaaatccccagcgaatacactgaagttacaagtgagtatgcaaggcaactgcgaggcctagcgagcaaaatactttcagcactatcaatttgcttgggattagaagaaggaaggctagagaaggaagttggtggcgtggaagagc

GaPDS-GaANR

aaatgctgacttggccagagaaagtgaaatttgcaataggactcctaccagcaatgctgggtggacaaccttatgttgaggcccaagatggtttatctgttaaagactggatgagaaagcagggcgtacctgatcgtgtgactgaggaggtgtaacactacagtcagggaccctgacaaccagaagaagatctctcaccttgtaacactacaagagttgggagacttgaagatctttcaggcggatttaactgatgaagggagctttgatgcccctattgctggttgtgaccttgtcttccatgttgcgacacccgttaactttgcttctgaagatccagaggatgac
