# Supplementary material for: Development of Agrobacterium-Mediated Virus-Induced Gene Silencing and Performance Evaluation of Four Marker Genes in Gossypium barbadense
Source: PLoS One. 2013 Sep 2;8(9):e73211. doi: 10.1371/journal.pone.0073211 (PMC3759462; doi:10.1371/journal.pone.0073211)
Supplement: File S2 — Extraction and assay of soluble and insoluble PA(s). (DOC) [file pone.0073211.s003.doc]

**Supplementary file 2. Extraction and assay of soluble and insoluble PA(s)**

The extraction of leaf PAs was carried out according to the method reported by Peng et al (2012) with minor modification. 100mg fresh materials were ground to fine powder by liquid nitrogen and transferred into a new tube, then 1 ml acetone were added into the tube immediately. Vortexed the tube for 30s, and centrifuged in 6,000 rpm for 3 min, and then acetone phase was transferred into new 5 ml tube. Repeated twice, then acetone phase was putted together. The residue should be collected for insoluble PAs assay. Dried acetone in vacuum completely, added 500μl ddH_2_O water and 200μl chloroform, vortexed for 30s and short centrifuged, chloroform phase was removed. The same manipulation was repeated twice to remove chlorophyll completely. Added 500μl ethyl acetate into water phase and vortexed for 30s and short centrifuged, and then ethyl acetate phase was transferred in a new tube. Repeated twice, and ethyl acetate phase was putted together. Dried ethyl acetate phase under reduced pressure in vacuum, and residue was dissolved in 100μl methanol.

For soluble PAs, each 50µl PAs extraction was mixed with 950µl butanol:HCl (95: 5, v/v). The mixture was then boiled for 1h. After cooling to approximately room temperature, A550nm OD value of each sample had been measured by spectrophotometer.

For insoluble PAs, the extracted residue was washed with 1 ml acetone twice time to remove chlorophyll and other flavonoids completely. Then the residue was mixed with 1 ml butanol:HCl (95: 5, v/v) in a tube and boiled for 1h. After cooling to room temperature, centrifuged the tube in 6,000 rpm for 3 min, and transferred liquid phase into a new tube, A550nm OD value of each sample had been measured by spectrophotometer.

Peng QZ, Zhu Y, Liu Z, Du C, Xie DY (2012) An integrated approach to demonstrating the ANR pathway of proanthocyanidin biosynthesis in plants. Planta 236:901-918
