# Supplementary material for: Development of Agrobacterium-Mediated Virus-Induced Gene Silencing and Performance Evaluation of Four Marker Genes in Gossypium barbadense
Source: PLoS One. 2013 Sep 2;8(9):e73211. doi: 10.1371/journal.pone.0073211 (PMC3759462; doi:10.1371/journal.pone.0073211)
Supplement: Table S1 — Primer sets used to isolate target gene fragment sequences for PCR and assay for qRT-PCR. (DOC) [file pone.0073211.s004.doc]

Table S1. Primer sets used to isolate target gene fragment sequences for PCR and assay for qRT-PCR

| Genes | Forward | Reverse |
| --- | --- | --- |
| For gene fragment sequences isolation | | |
| *5’-GaPDS* | gcc**gaattc**tgcgatgccaaataaacctg | gct**ggtacc**acacctcctcagtcactcg |
| *3’-GaPDS* | gcc**gaattc**gcctgaagactggagagag | gct**ggtacc**gctttactctgatctgcag |
| *GaCLA1* | gcc**gaattc**gccctttgtgcatcttcc | gctggtaccctctaggggcattgaag |
| *GaANR* | gcc**gaattc**aacactacagtcagggaccc | gct**ggtacc**tgcggcagatgatgtcaaga |
| *GaANS* | gcc**gaattc**gcttgagtgggaggactactt | gct**ggtacc**ttgggaacacatttagcggtc |
| For qRT-PCR analysis | | |
| *GaPDS* | ctatctagcaggtgattac | cccaaagtacaaagcaactc |
| *GaCLA1* | gtaaagtggaggccattgg | aagcacattgaacaccgttg |
| *GaANR* | ggtcgatatatatgcagtg | caacttgggtttggagggg |
| *GaANS* | gatctcatgggcagttttc | tatgctgagcaaaggtacg |
| *TRV RNA2* | ctaaatagggctaattgtg | tagtgtcgtcaagccacttcc |
| *GaUBI* | gagacgtagttagaaaggaag | agtacgttcccattccggaac |
